# Baseline Participant Characteristics in the DIAN‐TU‐001 Trial

**DOI:** 10.1002/alz70859_105299

**Published:** 2025-12-26

**Authors:** Jorge J. Llibre‐Guerra, Jin Zhou, Lon S. S Schneider, Erica Andreozzi, David B. Clifford, Sibabrata Banerjee, Guoqiao Wang, Eric McDade, Larisa Reyderman, Randall J. Bateman

**Affiliations:** ^1^ Washington University School of Medicine, St. Louis, MO USA; ^2^ Eisai Inc., Nutley, NJ USA; ^3^ Alzheimer’s Disease Research Center, Keck School of Medicine, University of Southern California, Los Angeles, CA USA; ^4^ Banner Alzheimer's Institute, Phoenix, AZ USA; ^5^ Keck School of Medicine of USC, Los Angeles, CA USA; ^6^ Washington University School of Medicine, St Louis, MO USA

## Abstract

**Background:**

Dominantly Inherited Alzheimer’s disease (DIAD) is a rare form of AD that has its onset typically at 30‐50 years old. DIAD has a small prevalence (∼1%), but shares pathophysiology with the more prevalent late‐onset sporadic form of AD. The aim of the Dominantly Inherited Alzheimer Network ‐Trials Unit (DIAN‐TU) is to find solutions to treat or prevent DIAD and to potentially apply these solutions to all forms of AD. Herein, we describe the baseline characteristics from the DIAN‐TU‐001 Tau NexGen arm evaluating the anti‐tau therapy etalanetug with background lecanemab therapy.

**Methods:**

DIAN‐TU‐001 is a phase II/III multicenter randomized, double‐blind, placebo‐controlled platform trial of potential disease modifying therapies utilizing biomarker, cognitive, and clinical endpoints in DIAD. The purpose of this study is to assess the safety, tolerability, biomarker, cognitive and clinical efficacy of investigational products in participants with an AD‐causing mutation by determining if treatment with the anti‐tau antibody (etalanetug) slows the rate of progression of cognitive/clinical impairment or improves disease‐related biomarkers. Baseline characteristics are summarized descriptively for both cohorts (Cohort 1: Symptomatic Population (CDR=0.5‐1); Cohort 2: Asymptomatic Population (CDR=0)).

**Results:**

As of a data cutoff of December 3, 2024, 243 participants were screened and 197 randomized in DIAN‐TU‐001 Tau NexGen trial (Cohort 1: 97; Cohort 2: 100). Baseline characteristics of Cohort 1 and Cohort 2 were similar except for cognitive values, all of which varied as expected per the differing CDR study entry criteria for the two cohorts. Mean age (standard deviation [SD]) was 47.8 (8.6) and 43.4 (8.4) years in Cohort 1 and 2, respectively. At baseline, mean CDR‐SB values (SD) were 3.7 (2.1) for Cohort 1 and 0.1 (0.3) for Cohort 2. APOE4 carrier frequency is consistent with that of the general population, with 25.8% and 21.0% carriers in Cohorts 1 and 2, respectively. The most common gene type in each group was PSEN1 (Cohort 1:78.4%; Cohort 2:85.0%).

**Conclusions:**

The baseline characteristics for DIAN‐TU‐001 Tau NexGen trial were consistent with clinical features of asymptomatic and symptomatic DIAD. These characteristics were also generally consistent between Cohort 1 and Cohort 2 with the exception of clinical, and cognitive scores.

